# Women with epilepsy in Rwanda—a real real‐world study

**DOI:** 10.1111/ene.16295

**Published:** 2024-04-03

**Authors:** Bernhard J. Steinhoff

**Affiliations:** ^1^ Kork Epilepsy Center Kehl‐Kork Germany; ^2^ Medical Faculty University of Freiburg Freiburg Germany

In recent years, it has become somewhat fashionable to perform and publish so‐called “real‐world” studies. Usually, these are non‐interventional observational and uncontrolled monocenter or multicenter studies dealing with the efficiency of recently introduced new generation antiseizure medications (ASMs) after labeling. But, is this real world really reflecting what we should call the real epilepsy world of 2024?

An original article of Dedeken et al. published in this issue of the *European Journal of Neurology* [1] investigated a sociologically most important real‐world question when 100 women with epilepsy (WWE) were interviewed in a cross‐sectional study performed at one hospital in Umuhoza, Rwanda. Of 100 WWE (age range 18–67 years), 48 had been pregnant, most of them (*n* = 39) after the initial diagnosis of epilepsy.

Due to the limited availability of ASM in Rwanda, first and second generation ASMs including valproic acid (VPA) were more widely used than would be the case for WWE of childbearing age in European countries. In fact, 46% of the WWE of reproductive age were on VPA. Interestingly, no congenital abnormalities were reported. Thirty‐nine WWE with previous pregnancies reported 91 pregnancies with a rate of early abortions of 14%.

Several concerning additional observations were made.

- About a quarter used contraception, 50% of whom were under treatment with enzyme‐inducing ASMs.
- Folic acid prophylaxis never happened prior to pregnancy.
- Adherence to ASMs was limited during pregnancy and breastfeeding periods.
- High rates of stigmatization and discrimination were reported.

Although only two WWE got advice prior to pregnancy and many pregnancies were carried out under the influence of VPA, no major malformations were reported. This certainly does not suggest that the teratogenic risk of valproate was negligible in the investigated group. In fact, the numbers of patients were too low to identify malformations with a reasonable probability. Another finding was the rather high frequency of seizure increases during pregnancy of 36% which supported the conclusion of unsatisfactory information since most of the seizure increases resulted from poor ASM adherence.

The major limitation of this important work is the rather low number of patients and the monocenter study design. One has to suggest that a nationwide registry (which is probably a non‐realistic goal) would reveal even more concerning outcome data than the pilot study published in this issue.

Still, the most important studies in clinical medicine are those that finally confirm with the help of scientific methods what everybody was convinced about already. One famous example is the paper of Patrick Kwan and Martin Brodie back in 2000 [2] or the SANAD trials concerning the efficacy of VPA in generalized and unclassified epilepsies [3, 4]. Every experienced epileptologist knew that drug‐resistant epilepsies are easy to identify early during ASM treatment and that VPA is extremely effective in generalized epilepsies but these studies finally confirmed that undoubtedly. The findings of the paper of Dedeken et al. [1] were predictable which in turn confirms its quality. Although the willingness to participate of both the investigated WWE and the participating hospital reflect a positive selection bias, still the results show the previously expected lack of education and knowledge that would help to improve the situation of WWE in subsaharan countries. Right now, we have to realize globally how dangerous a lack of education is. This lack of knowledge is certainly responsible for the extremely high rates of discrimination and stigma that are unveiled by the study of Dedeken et al. [1].

Finally, there are some economic facts that are difficult to change. The fancy new ASMs the so‐called real‐world studies deal with are not available in countries like Rwanda and will probably not be available in the next few years or even decades. Thus, so many people with epilepsy are not able to participate in the unquestionable progress epilepsy therapy has made recently. This is the ultimate tragedy this real real‐world study confirms again.

## AUTHOR CONTRIBUTIONS

**Bernhard Steinhoff:** Conceptualization; writing – original draft.

## CONFLICT OF INTEREST STATEMENT

Advisory and consulting honoraria: Angelini, Jazz/GW Pharmaceuticals, Precisis, Roche Diagnostics, UCB. Speaker‘s honoraria: Al Jazeera, Angelini, Bial, Desitin, Eisai, Jazz/GW Pharmaceuticals, Medscape, Tabuk, Teva, UCB, Zogenix. Research support: Eisai, European Union, Jannsen‐Cilag, Jazz/GW Pharmaceuticals, SK Life Sciences, UCB, Zogenix.

## Data Availability

The data that support the findings of this study are available on request from the corresponding author. The data are not publicly available due to privacy or ethical restrictions.
